# A dataset of factors affecting sustainable consumption intention in Vietnam

**DOI:** 10.1016/j.dib.2022.108127

**Published:** 2022-04-01

**Authors:** Le-Huy Tran, Ngoc-Anh Nguyen, Thi-Diu Tran, Thi-Phuong-Linh Nguyen

**Affiliations:** National Economics University, Faculty of Business Management, Department of Business Management, 207 Giai Phong, Hanoi, Vietnam

**Keywords:** Sustainable consumption intention, Theory of planned behavior, The norm activation model, Vietnam

## Abstract

The dataset explores the factors affecting the sustainable consumption intention of Vietnamese consumers. The research model was built based on a combination of the theory of planned behavior (TPB) and norm activation model (NAM) with 7 factors and 26 items. The data set was the result of a large-scale survey of 551 Vietnamese people with different demographic characteristics conducted in November 2021. The data set was the basis for identifying factors that influence the sustainable consumption intention of Vietnamese consumers, thereby making recommendations to state management agencies and enterprises to promote sustainable consumption intentions in Vietnam.

## Specification Table

**Table:** 

Subject	Social Science
Specific subject area	Sustainable consumption behavior, theory of planned behavior, norm activation model
Type of data	Table
How data were acquired	Design a questionnaire on Microsoft Word Survey using Google Form Storing survey data using Microsoft Excel
Data format	Raw Analyzed
Description of Data collection	The dataset has been collected from the online survey on sustainable consumption intentions of Vietnamese consumers in November 2021. The results of the collection of 551 valid questionnaires were included in the analysis.
Data source location	Region: Asia. Country: Vietnam.
Data accessibility	Mendeley Data Repository name: Sustainable Consumption Intention in Vietnam Data identification number: 10.17632/2n3sc78mph.2 Direct URL to data: https://data.mendeley.com/datasets/2n3sc78mph/2

## Value of the Data

• The dataset explores the relationship between factors affecting the sustainable consumption intention of Vietnamese consumers based on the TPB-NAM integrated model. At the same time, the data set also included demographic characteristics of respondents.
• The dataset applies and confirms the suitability of the scales in the TPB-NAM integrated model to study the sustainable consumption intention in Vietnam.
• Other researchers can use the dataset to compare sustainable consumption intention with similar studies in geographically different regions.

## 1 Data Description

Sustainable consumption is the use of goods and services that meet basic needs and provide a better quality of life, while reducing the use of natural resources, hazardous materials and waste emissions, so as not to endanger the needs of future generations [1]. A special focus of sustainable consumption is on the economic activity of selecting, using and disposing of goods and services for social and environmental benefits. Sustainable consumption intentions have been studied a lot in such areas as: online shopping [2], energy-saving appliances [3], green purchases [4,5], recycling [6]. Previous studies have only focused on sustainable consumption intentions and behavior for specific industries or products. In Vietnam, the concept of sustainable consumption is still quite new, there have not been many studies on this topic [7], especially using the TPB-NAM combined model to research sustainable consumption intentions. Therefore, the authors decided to do this paper, in order to test the relevance of this theory to the Vietnamese context. This study was built based on seven factors from the TPB-NAM integration model (intention, attitude, subject norms, perceived behavioral control, awareness of consequences, ascription of responsibility, personal norm). TPB explains one's intentional behavior stemming from personal expectations and interests, while NAM focuses on one's behavior stemming from altruistic and moral beliefs, specifically beliefs about right and wrong [8].

The dataset was collected through 2 survey parts: the first part explores the respondents' demographic characteristics including gender, age, education level, marital status, and income (Table 1), the second part explores respondents' consent to statements related to factors affecting sustainable consumption intention. Vietnamese consumers spent about 5 min filling out the survey and the authors received 551 valid responses.

**Table 1:** Respondents’ characteristics.

*Characteristics*	N	%
*Gender (GE)*	551	100
Male	228	41.4
Famale	323	58.6
*Age (AG)*	551	100
Under 18	65	11.8
18–24	376	68.2
25–35	51	9.3
36–45	28	5.1
46–55	22	4.0
Over 56	9	1.6
*Education level (EL)*	551	100
Haven't finished high school	26	4.7
High school	60	10.9
Intermediate college	27	4.9
University	407	73.9
After university	31	5.6
*Marital status (MS)*	551	100
Single	436	79.1
Married	82	14.9
Divorce	13	2.4
Others	20	3.6
*Monthly income (MI)*	551	100
Under 6	383	69.5
6–10	66	12.0
11–15	38	6.9
16–20	30	5.4
21–30	18	3.3
Over 30	16	2.9

## 2 Experimental Design, Material and Methods

The survey was conducted in both direct and online form in November 2021. In the form of direct data collection at some supermarkets, the authors listed the top 6 supermarkets in three major cities (Hanoi, Ho Chi Minh and Da Nang), where have a number of customers in Vietnam. Then, with the support of collaborators, the author conducted a survey at 7:30 p.m. – 9 p.m. every week at the exit of supermarkets. In the form of online data collection via Google Forms, the author made a list of a number of businesses that publish their employees' email addresses on the company's official website and randomly selected 10 businesses with diverse business fields, and then sent the survey link to these people. In addition, the authors asked for the help of 10 universities across the country to collect student email information, thereby contacting them through the provided email and requesting a survey. Each survey participant will receive a prize code and 15 randomly selected lucky people will receive a $10 phone card.

• Students: 415 answers accounting for 75.32% of the total valid answers, and 5 answers were not valid (randomly selected based on a list of personal information, which was gathered from a number of universities).
• Working people: 30 answers accounting for 5.44% of the total valid answers, and 1 answer was not valid (randomly selected based on contact information, which is searched on some websites of enterprises, government agencies).
• Consumers: 106 answers accounting for 19.24% of the total valid answers, and 3 answers were not valid (randomly selected at the supermarket direct survey).

**Fig. 1:**
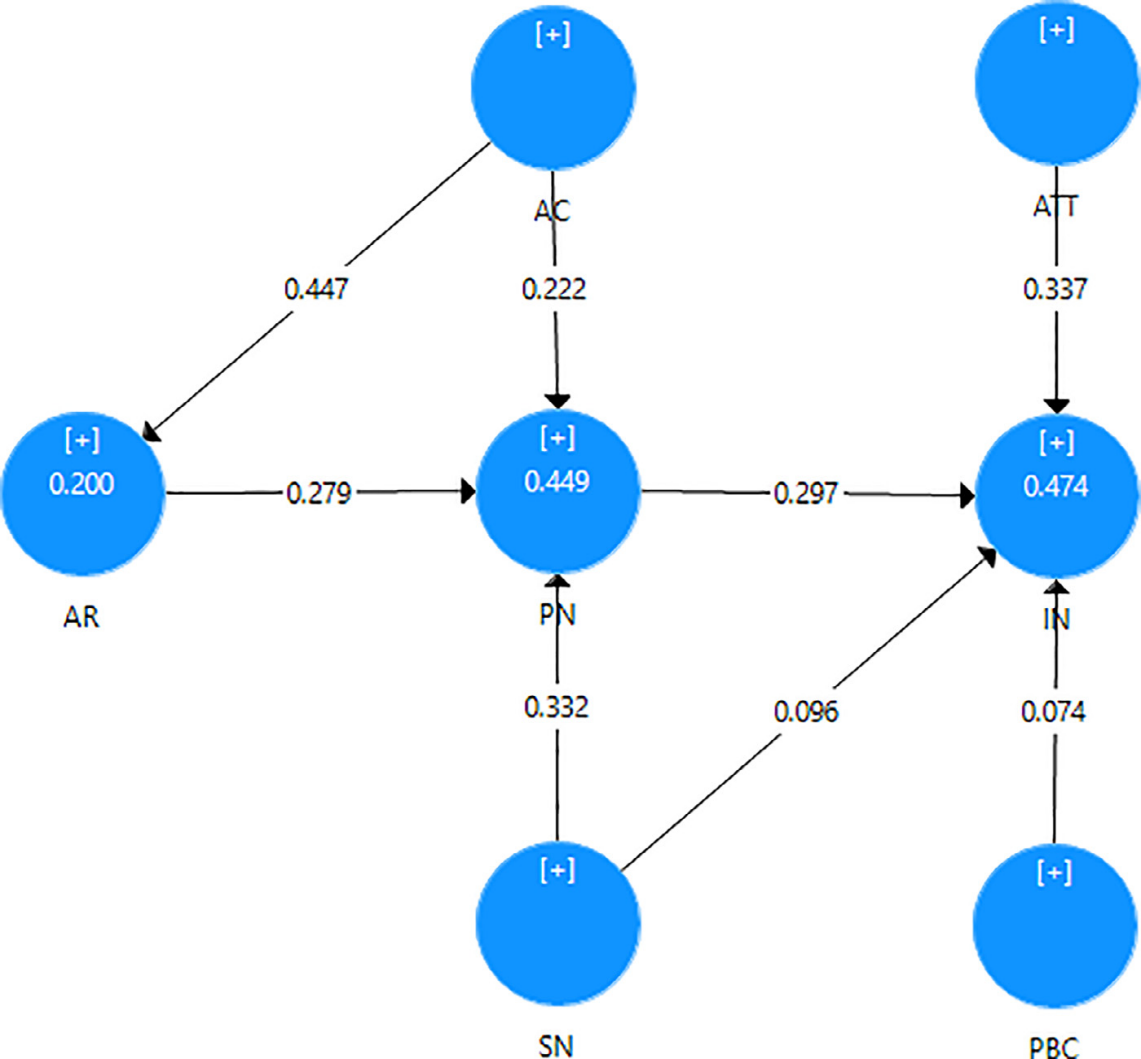
Structural Equation Modeling. The results of the test show that the factors positively affect each other, though more or less; except PBC has no effect on IN because p-value = 0.057 > 0.05. ATT is the strongest factor affecting IN with β = 0.337.

This survey was designed by the authors with 26 items with 5 demographic characteristics, designed based on a 5-likert scale (1: strongly disagree, 2: disagree, 3: neutral, 4: agree, 5: strongly agree), focusing on 7 factors: intention, attitude, subject norms, perceived behavioral control, awareness of consequences, ascription of responsibility, personal norm. All items are based on previous studies [9,10]. Before the analysis, the variables were encoded and the data were checked to ensure the validity of each questionnaire. The results of data collection shown that 600 answer sheets were collected, including 551 valid answer sheets (91.83%). All responses were imported into SPSS 22 and Smart PLS 3 were used to analyze the data. The data were analyzed by descriptive statistics (Table 2), Cronbach's Alpha reliability test, EFA exploratory factor test (Table 3), Person correlation test (Table 4), Structural Equation Modeling (Fig. 1).

**Table 2:** Description results of participants’ responses.

Variables		N	Min	Max	Mean	Std. Deviation
ATT1	I care about the quality of the environment where I live (e.g. water, clean air, land, forests, etc.).	551	1.0	5.0	4.160	0.8800
ATT2	I support environmentally friendly products (recycled materials, green label products).	551	1.0	5.0	4.216	0.8835
ATT3	I am willing to reuse plastic, bottles and paper items.	551	1.0	5.0	4.054	0.8360
ATT4	I support increased use of renewable energy sources.	551	1.0	5.0	4.220	0.8162
ATT5	I am willing to participate in programs that promote sustainable consumption.	551	1.0	5.0	3.946	0.8446
IN1	I want to choose sustainable products.	551	1.0	5.0	3.949	0.8807
IN2	I want to use sustainable products.	551	1.0	5.0	3.971	0.8734
IN3	I want to practice environmentally friendly behavior (sorting, recycling, saving energy, etc.).	551	1.0	5.0	4.011	0.8570
IN4	I will convince everyone to practice sustainable consumption behavior.	551	1.0	5.0	3.788	0.9187
PN1	I believe that I am responsible for sustainable consumption.	551	1.0	5.0	3.902	0.8787
PN2	I feel an obligation to comply with sustainable production and consumption regulations.	551	1.0	5.0	3.882	0.8773
PN3	Choosing sustainable products is in line with my ethical principles.	551	1.0	5.0	3.773	0.8910
PN4	My personal values (personality, habits, …) encourage me to choose sustainable consumption.	551	1.0	5.0	3.820	0.8997
AC1	The non-sustainable consumption is the cause of air and water pollution; climate change and resource depletion.	551	1.0	5.0	3.918	0.9566
AC2	The lack of sustainable consumption is the cause of wildlife extinction.	551	1.0	5.0	3.813	0.9589
AC3	Non-sustainable consumption affects people's mental and physical health.	551	1.0	5.0	3.902	0.9674
SN1	My family members think that I should consume sustainably.	551	1.0	5.0	3.672	0.8152
SN2	People around me (friends, colleagues, neighbors) think that I should consume sustainably.	551	1.0	5.0	3.641	0.8145
SN3	My significant other (idols,…) think that I should consume sustainably.	551	1.0	5.0	3.695	0.8579
SN4	Society and community advocate sustainable lifestyles.	551	1.0	5.0	3.838	0.8187
PBC1	I will still consume sustainably even if people tell me not to.	551	1.0	5.0	3.800	0.9342
PBC2	I can easily control sustainable consumption behavior.	551	1.0	5.0	3.695	0.9561
PBC3	I have enough ability and knowledge for sustainable consumption.	551	1.0	5.0	3.699	0.9890
AR1	I feel responsible for the problems that arise from not practicing sustainable consumption.	551	1.0	5.0	3.764	0.9178
AR2	I see environmental problems if I don't practice sustainable consumption.	551	1.0	5.0	3.837	0.9374
AR3	I believe that everyone has a responsibility to practice sustainable consumption.	551	1.0	5.0	3.967	0.9199

**Table 3:** Cronbach's alpha & explore factor analysis.

Variables	Items	Cronbach's Alpha	1	2	3	4	5	6	7
ATT α=0.916	ATT2	0.895	0.779						
	ATT4	0.899	0.753						
	ATT1	0.892	0.753						
	ATT5	0.899	0.670						
	ATT3	0.900	0.654						

IN α=0.950	IN1	0.925		0.844					
	IN2	0.924		0.844					
	IN3	0.935		0.818					
	IN4	0.953		0.804					

PN α=0.923	PN3	0.900			0.780				
	PN1	0.898			0.780				
	PN2	0.904			0.757				
	PN4	0.898			0.745				

SN α=0.869	SN2	0.811				0.804			
	SN1	0.828				0.763			
	SN3	0.833				0.694			
	SN4	0.857				0.678			

AC α=0.922	AC2	0.876					0.877		
	AC3	0.901					0.862		
	AC1	0.886					0.861		

PBC α=0.888	PBC2	0.820						0.866	
	PBC3	0.821						0.807	
	PBC1	0.878						0.770	

AR α=0.897	AR1	0.860							0.819
	AR2	0.844							0.789
	AR3	0.854							0.773

**Table 4:** Person correlations test.

		GE	AG	EL	MS	MI	IN	ATT	SN	PBC	PN	AR	AC
**GE**	Pearson Correlation	1											
	Sig												

**AG**	Pearson Correlation	−0.185⁎⁎	1										
	Sig	.000											

**EL**	Pearson Correlation	−0.053	.112⁎⁎	1									
	Sig	.211	.008										

**MS**	Pearson Correlation	−0.093*	.393⁎⁎	−0.154⁎⁎	1								
	Sig	.029	.000	.000									

**MI**	Pearson Correlation	−0.207⁎⁎	.465⁎⁎	.162⁎⁎	.237⁎⁎	1							
	Sig	.000	.000	.000	.000								

**IN**	Pearson Correlation	−0.069	.192⁎⁎	.527⁎⁎	.013	.256⁎⁎	1						
	Sig	.108	.000	.000	.761	.000							

**ATT**	Pearson Correlation	−0.034	.105*	.386⁎⁎	−0.012	.150⁎⁎	.621⁎⁎	1					
	Sig	.422	.013	.000	.776	.000	.000						

**SN**	Pearson Correlation	.014	.043	.358⁎⁎	−0.008	.119⁎⁎	.523⁎⁎	.651⁎⁎	1				
	Sig	.738	.313	.000	.859	.005	.000	.000					

**PBC**	Pearson Correlation	.020	.128⁎⁎	.367⁎⁎	−0.022	.144⁎⁎	.456⁎⁎	.506⁎⁎	.538⁎⁎	1			
	Sig	.636	.003	.000	.609	.001	.000	.000	.000				

**PN**	Pearson Correlation	−0.028	.131⁎⁎	.395⁎⁎	−0.009	.136⁎⁎	.602⁎⁎	.626⁎⁎	.565⁎⁎	.536⁎⁎	1		
	Sig	.518	.002	.000	.841	.001	.000	.000	.000	.000			

**AR**	Pearson Correlation	.008	.088*	.358⁎⁎	−0.031	.110⁎⁎	.493⁎⁎	.597⁎⁎	.541⁎⁎	.452⁎⁎	.557⁎⁎	1	
	Sig	.850	.039	.000	.473	.010	.000	.000	.000	.000	.000		

**AC**	Pearson Correlation	−0.045	.044	.228⁎⁎	−0.055	.086*	.409⁎⁎	.484⁎⁎	.377⁎⁎	.350⁎⁎	.471⁎⁎	.446⁎⁎	1
	Sig	.294	.303	.000	.195	.044	.000	.000	.000	.000	.000	.000	

⁎⁎ . Correlation is significant at the 0.01 level (2-tailed).

⁎ . Correlation is significant at the 0.05 level (2-tailed).

## Ethics Statement

The authors kept to all ethical concerns during the data gathering process. The authors got the consent of the respondent when conducting surveys and ensured that all information was used for research purposes and was absolutely confidential. The study was not conducted in accordance with the Declaration of Helsinki.

## CRediT Author Statement

**Le-Huy Tran:** Writing – original draft preparation, Writing – review & editing; **Ngoc-Anh Nguyen:** Software, Formal analysis, Data Curation; **Thi-Diu Tran:** Conceptualization, Visualization, Investigation; **Thi-Phuong-Linh Nguyen:** Supervision, Methodology.

## Declaration of Competing Interest

The authors declare that they have no known competing financial interests or personal relationships that could have appeared to influence the work reported in this paper.

## Data Availability

Sustainable Consumption Intention in Vietnam (Original data) (Mendeley Data).
